# White-Matter Integrity and Working Memory: Links to Aging and Dopamine-Related Genes

**DOI:** 10.1523/ENEURO.0413-21.2022

**Published:** 2022-04-13

**Authors:** Xin Li, Alireza Salami, Bárbara Avelar-Pereira, Lars Bäckman, Jonas Persson

**Affiliations:** 1 Aging Research Center, Karolinska Institute and Stockholm University, 171 65 Solna, Sweden; 2 Umeå Center for Functional Brain Imaging (UFBI), Umeå University, 901 87 Umeå, Sweden; 3 Wallenberg Centre for Molecular Medicine, Umeå University, 901 87 Umeå, Sweden; 4 Department of Psychiatry and Behavioral Sciences, School of Medicine, Stanford University, Stanford, California 94305-5101; 5 Center for Lifespan Developmental Research (LEADER), School of Law, Psychology and Social Work, Örebro University, 701 82 Örebro, Sweden

**Keywords:** aging, *COMT*, dopamine, DTI, white matter, working memory

## Abstract

Working memory, a core function underlying many higher-level cognitive processes, requires cooperation of multiple brain regions. White matter refers to myelinated axons, which are critical to interregional brain communication. Past studies on the association between white-matter integrity and working memory have yielded mixed findings. Using voxelwise tract-based spatial statistics analysis, we investigated this relationship in a sample of 328 healthy adults from 25 to 80 years of age. Given the important role of dopamine (DA) in working-memory functioning and white matter, we also analyzed the effects of dopamine-related genes on them. There were associations between white-matter integrity and working memory in multiple tracts, indicating that working-memory functioning relies on global connections between different brain areas across the adult life span. Moreover, a mediation analysis suggested that white-matter integrity contributes to age-related differences in working memory. Finally, there was an effect of the *COMT* Val158Met polymorphism on white-matter integrity, such that Val/Val carriers had lower fractional anisotropy values than any Met carriers in the internal capsule, corona radiata, and posterior thalamic radiation. As this polymorphism has been associated with dopaminergic tone in the prefrontal cortex, this result provides evidence for a link between DA neurotransmission and white matter. Together, the results support a link between white-matter integrity and working memory, and provide evidence for its interplay with age- and DA-related genes.

## Significance Statement

Working memory, a core function underlying many higher-level cognitive processes, requires cooperation of multiple brain regions. Using tract-based spatial statistics analysis, we found associations between white-matter integrity and working memory in multiple tracts in a sample of 328 healthy participants from 25 to 80 years old. This result indicates that working-memory functioning relies on global connections between different brain areas across the adult life span. Moreover, a novel voxelwise mediation analysis suggested that white-matter integrity contributes to age-related differences in working memory, with the strongest effect being observed in the corpus callosum and superior longitudinal fasciculus. Finally, we found an effect of the *COMT* Val158Met polymorphism on white-matter integrity, providing evidence for a link between DA neurotransmission and white matter.

## Introduction

Working memory (WM) refers to a capacity-limited system, in which information is maintained for short periods of time and manipulated or updated to achieve task-related goals (Baddeley, 1986, 1992). Successful WM performance relies on the interaction among multiple cognitive processes and has been linked to engagement of a widespread network of brain regions, such as the frontoparietal network (Kastner and Ungerleider, 2000; Curtis and D’Esposito, 2003; Koenigs et al., 2009) and basal ganglia (O’Reilly, 2006; Dahlin et al., 2008; McNab and Klingberg, 2008). Other brain regions, such as visual cortex and temporal regions may also be involved when visual or semantic stimuli are encoded and maintained in WM (Ranganath et al., 2004; Axmacher et al., 2007).

Diffusion tensor imaging (DTI) assesses *in vivo* white-matter integrity, which might reflect the efficiency of communication among brain networks. A few DTI studies have reported associations between white-matter integrity and WM performance (Deary et al., 2006; Davis et al., 2009; Kennedy and Raz, 2009a; Charlton et al., 2008, 2010b). However, their effect sizes are generally small, and there are inconsistencies regarding the localization of the effects. Moreover, it remains unclear whether the associations between white matter and WM are localized or rather are widespread across multiple tracts, and whether the associations vary across the entire brain (i.e., follow an anterior–posterior or inferior r–superior gradient). Functional magnetic resonance imaging (fMRI) studies have reported the strongest brain activation in the most demanding WM conditions (Veltman et al., 2003; Nagel et al., 2011). It is therefore possible that more demanding WM tasks require stronger white-matter connectivity to facilitate interaction between distal brain regions. There is no study to date exploring the link between different levels of WM demand and white matter.

Relationships of white-matter integrity to other cognitive domains, such as episodic memory and verbal ability, are typically weak (Vernooij et al., 2009; Salami et al., 2012). However, strong associations with processing speed have been demonstrated (Salami et al., 2012; Lövdén et al., 2014; Kuznetsova et al., 2016). There is a substantial shared variance between WM and processing speed (Oberauer et al., 2000; Ackerman et al., 2002), although it remains unknown whether potential white matter–WM associations are, at least partly, independent of processing speed.

Many previous studies have shown WM impairment with advancing age (Hultsch et al., 1992; Park et al., 2002). The underlying cause of age-related deficits is not fully understood. One previous study showed that age-related changes in global white-matter integrity explained 11% of the age-related WM decline (Charlton et al., 2010a). It remains unknown which white-matter tracts might account for age-related impairment in WM. Using a novel approach, voxelwise mediation analysis, the current study aimed to delineate the whole-brain pattern of white-matter integrity in relation to age-related differences in WM.

Dopamine (DA) plays a critical role in WM (Brozoski et al., 1979; Landau et al., 2009; Cools and D’Esposito, 2011) and has been associated with white-matter integrity (Rieckmann et al., 2016). Several genetic polymorphisms have been related to DA receptor density in cortical and subcortical brain regions, including *DRD2/ANKK1* Taq1A, *DRD2-*C957T, and *COMT*-Val158Met (Lotta et al., 1995; Thompson et al., 1997; Pohjalainen et al., 1998; Jönsson et al., 1999; Hirvonen et al., 2004, 2009a,b). These polymorphisms have been associated with WM functioning (Xu et al., 2007; Berryhill et al., 2013; Nyberg et al., 2014). For example, carriers of the *COMT* Met allele, which has been associated with higher DA levels in the prefrontal cortex (PFC; Lotta et al., 1995), had greater white-matter integrity (Papenberg et al., 2015), and had higher efficiency in the dorsolateral PFC during WM performance (Nyberg et al., 2014) compared with Val carriers. The genetic effects were more pronounced in older compared with younger adults (Papenberg et al., 2015; Persson et al., 2015; Li et al., 2019), which is consistent with the resource modulation hypothesis. This hypothesis asserts that the relationship of brain resources to cognitive performance is nonlinear: genetic effects are thought to be larger when brain resources decrease, such as in old age (Lindenberger et al., 2008). To our knowledge, only two studies have shown the genetic effects of *COMT* (Papenberg et al., 2015) and C957T (Markett et al., 2017) on white matter; the effect of Taq1A has not been examined.

The main questions addressed in this study are as follows. (1) Is WM associated with white-matter integrity? (1a) If so, what is the spatial pattern of these associations? (1b) Do the associations change across WM load? And (1c) are the associations independent of the effects of processing speed? (2) Does white-matter integrity contribute to age differences in WM? And (3) do three DA-related genes, *DRD2/ANKK1* Taq1A, *DRD2*-C957T, and *COMT*-Val158Met, affect white-matter integrity, WM performance, and the associations between white matter and WM? These questions were investigated for the first time using a voxelwise whole-brain approach across the adult life span.

## Materials and Methods

### Participants

The current sample is drawn from a longitudinal population-based study, the Betula project (Nilsson et al., 1997, 2004). This project uses an age-homogeneous, narrow age cohort (NAC) design, where chronological age is held constant for each cohort (e.g., participants were recruited at ages 35, 40, 45). The purpose of using a NAC design is to decrease confounding effects introduced by different age cohorts, such as education and nutritional level (Rönnlund et al., 2005; Sternäng et al., 2008). The initial fMRI sample included 372 participants. Twenty participants were excluded because of a diagnosis of dementia (4 participants), stroke (10 participants), epilepsy (2 participants), Parkinson’s disease (1 participant), multiple sclerosis (1 participant), and hydrocephalus (2 participants). Study participants provided written informed consent, and the protocol was approved by the Ethical Review Board in Umeå.

Dementia status was assessed at baseline and reassessed every 5 years using a three-step procedure, according to the *Diagnostic and Statistical Manual of Mental Disorders*, fourth edition (American Psychiatric Association, 1994). First, an overall evaluation was performed by an examining physician. The diagnosis was then compared with a second independent diagnosis based on scores from several cognitive tests. In cases of disagreement, a supervising physician made a third and final diagnosis. The cognitive assessment used in the diagnoses included the Mini-Mental State Examination (MMSE; Folstein et al., 1975), the Clock test (Manos and Wu, 1994), and tests of episodic memory, working memory, processing speed, semantic memory, and fluid intelligence. The WM task of primary interest in this study was not used for diagnostic purposes.

Nine additional participants were excluded because of deviant brain morphology (three participants), head surgery (two participants), and vascular brain lesions and infarcts (four participants). Three participants without complete DTI data and 12 subjects with measurement artifacts were also excluded. The final sample consisted of 328 participants. All participants’ MMSE scores were ≥24, and their cognitive test results were within normal ranges for their age cohort. Thirteen participants without genetic data, 12 participants without WM performance data, and 6 participants without processing speed data were excluded from the corresponding analyses.

### Cognitive measurements

In the Betula dataset, the cognitive test battery covered a wide range of domains, including working memory, processing speed, fluid intelligence, verbal fluency, and episodic memory (Pedersen et al., 2021). The data for fluid intelligence, verbal fluency, and episodic memory have been reported previously (Salami et al., 2012). The current study focused on WM and processing speed.

#### Working memory

An in-scanner WM task was used that included the following three conditions with different cognitive loads: manipulation, maintenance, and control. Note that in the current study, WM load refers to different levels of cognitive demand, and not necessarily to the number of items kept in WM. In the manipulation condition, participants were shown two target letters and instructed to generate and keep the subsequent letters in the alphabet in memory. After a fixation star, a probe letter with a question mark was presented and participants were asked to decide whether the probe letter was a letter that occurred subsequent to any of the two target letters in the alphabet. In the maintenance condition, participants were shown four target letters and were asked to keep these letters in memory. The task was to decide whether the probe letter was the same as any of the target letters. In the control condition, four identical letters were presented, and participants were instructed to decide whether the probe letter was the same as the target letter. Accuracy in the three conditions was used to index WM performance under the three loads.

#### Processing speed

Participants undertook the following three processing speed tests: letter-digit substitution, pattern comparison, and letter comparison (Nilsson et al., 1997). For letter-digit substitution, participants were shown letters on paper and were required to write down the paired digits for each letter according to a letter-digit transformation key shown on the top of the paper. The score was the number of correct digits that the participant managed to fill in during 1 min (maximum = 125 digits). In the pattern-comparison task, participants were instructed to compare pairs of abstract line figures during 30 s (maximum = 30 figures). The letter-comparison task involved comparing pairs of nonword strings of three to nine letters and judging whether they were the same or different. The score was the number of correctly judged pairs during 30 s (maximum = 21 pairs). We used the sum of the *z*-transformed scores of the three processing speed tests in the following analyses, as this increases reliability and allows for a more accurate estimation of the construct under investigation.

### Genotyping

All participants from the initial fMRI sample underwent DNA extraction and genotyping of the candidate genes. Genotyping was performed on a platform described previously (Kauppi et al., 2011). Primers for PCR amplification were designed using the Sequenom MassARRAY System Designer software. Participants with a sample call rate of <0.9 or indications of genotyping errors were excluded. The genotype distributions for all three polymorphisms did not deviate from Hardy–Weinberg equilibrium (χ = 0.75, 2.18, and 0.05; *r* = 0.19, 0.56, and 0.47 for *DRD2/ANKK1*-Taq1A, *DRD2*-C957T, and *COMT*-Val168Met, respectively).

### Image acquisition and preprocessing

DTI data were collected on a 3 T scanner (Discovery MR750, General Electric) with a 32-channel head coil. Diffusion-weighted data with a spatial resolution of 0.98 × 0.98 × 2 mm were acquired by a single-shot, spin-echoplanar, T2-weighted sequence. The sequence parameters were as follows: TR = 8.0 s; 64 slices with no gap in between; 256 × 256 matrix (FOV = 250 mm); 90° flip angle; TE = 84.4 ms; three repetitions of 32 independent directions; b = 1000 s/mm^2^; and six b = 0 images. The experimental design consisted of three identical DTI sessions. All participants were examined on the same scanner with no software or hardware update during the data collection period.

Diffusion-weighted imaging (DWI) data were analyzed using the University of Oxford Center for Functional Magnetic Resonance Imaging of the Brain (FMRIB) Software Library (FSL) package (http://www.fmrib.ox.ac.uk/fsl). Before image preprocessing, three sessions of the subject-specific diffusion acquisitions were concatenated into a 4D file for each subject. Then the raw images were corrected for eddy current-induced distortions and head movements by affine aligning them to the first no-diffusion-weighted image (b = 0). The transformation matrix was then used to rotate bvec files (Jenkinson and Smith, 2001). A binary brain mask was generated using the first no-DWI with the Brain Extraction Tool to exclude nonbrain voxels. Finally, the preprocessed DWI files were fitted to the DTI model. The tensor matrix with information in three directions (eigenvalues) was obtained for each voxel within the brain mask. Voxelwise maps of fractional anisotropy (FA), mean diffusivity (MD), radial diffusivity (RD), and axial diffusivity (AD) were generated using the three eigenvalues.

### Tract-based spatial statistics

We used tract-based spatial statistics (TBSS; Smith et al., 2006) to analyze the FA/MD/AD/RD images. First, all FA images were transformed to Montreal Neurologic Institute (MNI) space, using the high-resolution standardized image (FMRIB158_FA) as a target. This method only applies registration once per subject and provides highly accurate realignment. Next, the transformed FA images were averaged to produce a mean FA image for the entire group. A single 4D file was created by merging all transformed FA images. The mean FA image was fed into the tract-skeleton generation program to produce a white-matter tract skeleton, which represents the white-matter tracts common to all subjects. Then a binary skeleton mask was created by thresholding the mean FA skeleton image with FA values >0.2. Finally, each subject’s FA image was projected onto the group skeleton mask. MD/RD/AD images were processed using a similar method as for FA images. Voxelwise statistics were then run on the skeletonized images. The current study focuses mainly on FA, which is the most commonly used DTI variable.

### Statistical analyses

For each DTI measurement, general linear models (GLMs) were fitted to the data to investigate the relationship of white-matter integrity to cognitive performance and genetic factors. For each model, the Randomise toolbox in FSL was used to assess the regression coefficients for each voxel and to generate *t* statistic maps. Threshold-free cluster enhancement (TFCE) was then applied on the *t* maps. The TFCE algorithm uses sensitive cluster-based inference without a need for a primary cluster-forming threshold. Because the null distribution of the TFCE output was unknown, a nonparametric permutation test was used to build up the null distribution, and to calculate corrected *p*-values for each voxel. Five thousand permutations were performed for each contrast, resulting in a minimum possible *p*-value of 0.0002 (1/*p*). An FWE-corrected *p*-value of <0.05 was considered statistically significant.

#### Associations between white-matter indices and WM

We first examined the associations between FA and the three WM conditions with age and sex as nuisance variables. WM load effects were examined by including pairs of the WM conditions (manipulation, maintenance, and control) in GLMs. The correlation coefficients were compared among the three conditions for each voxel. We included processing speed performance as an additional covariate in the models to investigate whether potential FA–WM associations were independent of speed. To explore the spatial pattern of FA–WM links, the *t* statistics were averaged across all voxels within the skeleton for each slice along the anterior–posterior or inferior–superior axis.

#### Voxelwise mediation analysis

We investigated whether white-matter integrity mediated the age effects on WM and the spatial pattern of these potential mediation effects using a voxelwise approach (Lett et al., 2017). We first examined the associations between age and white-matter indices using GLM and created a set of masks that demonstrated significant age effects (only negative age effects on white matter were observed). The following mediation analysis was conducted within the masks. The mediation effect was estimated using a Sobel test for each voxel (Eq. 1 below), in which *a* and *b* are two coefficients of the paths (from age to white matter and from white matter to WM) and *S_a_* and *S_b_* are the SDs of the coefficients. The voxelwise mediation analysis was conducted using the TFCE-mediation package (Lett et al., 2017) in Python (https://github.com/trislett/TFCE_mediation). First, the covariate (sex) was regressed out for the independent variable (age), the dependent variables (WM), and for the mediator (white-matter integrity). After the Sobel *z* values were calculated for each voxel using Equation 1, TFCE was applied on the *z* map. Then 5000 permutations were conducted on the resulting *z* images. FWE-corrected *p*-values were calculated based on the permutation distribution.

We compute the change in effect sizes before and after mediation. First, a regression model of age and sex on WM was fitted to estimate the total variance of WM explained by age (i.e., *r*^2^ before mediation). The FA value with the maximal mediation effects was extracted for each participant. Then both the FA values extracted from the last step, age and sex, were included in a second-step regression model. This was done to evaluate the total WM variance accounted for by both FA and age (i.e., *r*^2^ after mediation). The change in effect was calculated by (*r*^2^ after mediation – *r*^2^ before mediation)/*r*^2^ before mediation, as follows:

(1) $$z_{\text{value}}=\frac{a\text{×}b}{\sqrt{b^{2}\text{×}s_{a}^{2}+a^{2}\text{×}s_{b}^{2}+s_{a}^{2}\text{×}s_{b}^{2}}}.$$

#### Genetic effects

We examined the effects of the *DRD2/ANKK1*-Taq1A, *DRD2*-C957T, and *COMT* Val158Met polymorphisms on WM performance and white matter integrity by comparing the genetic groups, controlling for age and sex using GLMs. The slopes of the relation between white-matter and WM performance were then compared among genetic groups, with age and sex as covariates. To further investigate whether the genetic effect is magnified in older adults, we separated the whole sample into younger (≤60 years) and older (>60 years) age groups. This was done to ensure that the two age groups were as similar as possible in sample size (*n* = 34, 42, 73; and 35, 45, 86 for ValVal, MetMet, and ValMet in younger and older groups, respectively). Analysis of covariance was used to investigate age × gene interactions.

## Results

Demographic information is shown in 10 year intervals to illustrate the age distribution (Table 1), which was skewed toward older participants. There is a significant age × condition interaction (*F*_(7.65,474.28)_ = 13.22, *p* < 0.0001, η^2^ = 0.18). Follow-up tests revealed that the effects of conditions were observed in age groups >55 years, in which WM performance in manipulation was worse than in maintenance and control conditions (*p* values < 0.001 for all comparisons). Demographic information across genotypes for the three polymorphisms is shown in Table 2. No genetic effects were observed for WM performance, processing speed, or any demographic variables (*p* values > 0.1).

**Table 1:** Demographic information across age groups

	25–30 years (*n* = 18)	35–40 years (*n* = 18)	45–50 years (*n* = 19)	55–60 years (*n* = 100)	65–70 years (*n* = 104)	75–80 years (*n* = 89)
Sex (female/male)	9/9	9/9	10/9	48/52	54/50	43/26
Education, years	15.1 (2.1)	15.9 (2.7)	13.7 (2.6)	14.4 (3.2)	12.6 (4.3)	9.5 (3.5)
MMSE	28.2 (1.4)	28.9 (1.1)	28.6 (1.3)	28.2 (1.4)	28 (1.6)	27.6 (1.6)
Manipulation, accuracy	16.8 (1)	17 (1.2)	16.8 (0.9)	16.1 (1.6)	15 (2.9)	12.5 (3.7)
Maintenance, accuracy	17.6 (0.8)	17.8 (0.4)	17.6 (0.7)	17.2 (1.3)	16.7 (1.8)	15.8 (2.8)
Control, accuracy	17.9 (0.2)	17.7 (0.6)	17.8 (0.4)	17.7 (0.6)	17.3 (1.6)	16.8 (1.9)
Processing speed, accuracy	2.7 (2.5)	2.3 (3.1)	2.3 (1.9)	0.9 (2)	−0.6 (1.9)	−2.4 (2)

Values are given as mean (SD).

**Table 2:** Genotype-specific demographic information

*DRD2/ANKK1*-Taq1A	GG (*n* = 208)	GA (*n* = 93)	AA (*n* = 14)	Statistics
Age	62 (13.2)	60.1 (14.4)	60.7 (13.6)	1.04^*a*^
Sex (female/male)	118/90	47/46	4/10	4.7^*b*^
Education, years	13 (4.1)	12.8 (4.1)	13.4 (4)	0.002^*a*^
MMSE	28.1 (1.5)	28.1 (1.4)	28.2 (1)	0.003^*a*^
Manipulation, accuracy	15.1 (3)	15.3 (2.7)	15 (2.7)	0.11^*a*^
Maintenance, accuracy	16.8 (1.9)	16.9 (1.9)	16.7 (1.8)	0.001^*a*^
Control, accuracy	17.4 (1.4)	17.4 (1.2)	17.4 (1.4)	0.015^*a*^
Processing speed, accuracy	−0.1 (2.4)	0.3 (3)	0.2 (2.3)	1.22^*a*^

*DRD2*-C957T	TT (*n* = 93)	CT (*n* = 168)	CC (*n* = 54)	Statistics
Age	60.9 (14)	60.9 (14)	62 (12.6)	0.52^*a*^
Sex (female/male)	53/40	92/76	24/30	2.34^*b*^
Education, years	13 (4.1)	12.8 (4.2)	13.3 (3.9)	0.16^*a*^
MMSE	28.3 (1.6)	28.1 (1.4)	27.9 (1.4)	0.35^*a*^
Manipulation, accuracy	15.5 (3.1)	15 (2.8)	15 (2.6)	1.16^*a*^
Maintenance, accuracy	16.9 (2)	16.8 (2.1)	16.8 (1.5)	0.04^*a*^
Control, accuracy	17.4 (1.4)	17.4 (1.5)	17.4 (1.2)	0.007^*a*^
Processing speed, accuracy	−0.1 (2.8)	−0.02 (2.5)	0.35 (2.4)	0.008^*a*^

*COMT*-Val168Met	ValVal (*n* = 69)	ValMet (*n* = 159)	MetMet (*n* = 87)	Statistics
Age	61.6 (12.9)	61.5 (13.5)	61.6 (14.2)	0.001^*a*^
Sex (female/male)	37/32	89/70	43/44	0.97^*b*^
Education, years	13.1 (3.4)	13 (4.2)	12.7 (4.3)	0.19^*a*^
MMSE	28.1 (1.4)	28.1 (1.5)	28.1 (1.5)	0.11^*a*^
Manipulation, accuracy	15 (2.8)	15.2 (3.1)	15.2 (2.7)	0.032^*a*^
Maintenance, accuracy	17 (1.4)	16.8 (2.1)	16.8 (1.9)	<0.001^*a*^
Control, accuracy	17.5 (1)	17.4 (1.5)	17.4 (1.1)	0.071^*a*^
Processing speed, accuracy	0.1 (2.6)	0.2 (2.7)	−0.3 (2.5)	2.1^*a*^

Values are given as mean (SD).

^a^*F* value.

^b^χ^2^ value.

### White-matter microstructure and WM performance

**Figure 1.**
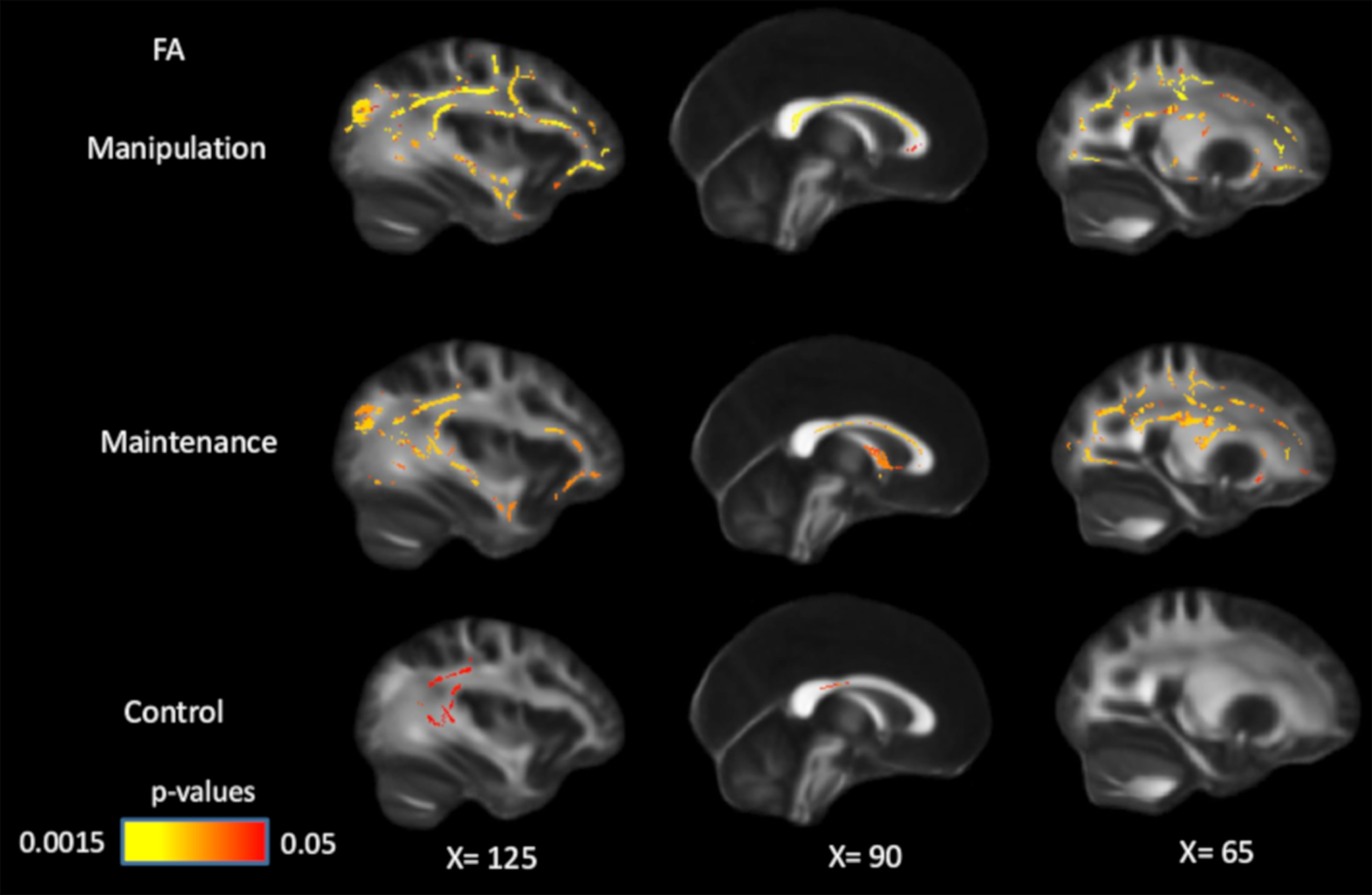
Positive associations between FA and WM performance in manipulation, maintenance, and control conditions, controlling for age and sex. Significant voxels (*p* < 0.05) are overlaid on the T1-weighted image, with yellow color indicating lower *p* values. Coordinates are given according to the MNI152 template.

**Figure 2.**
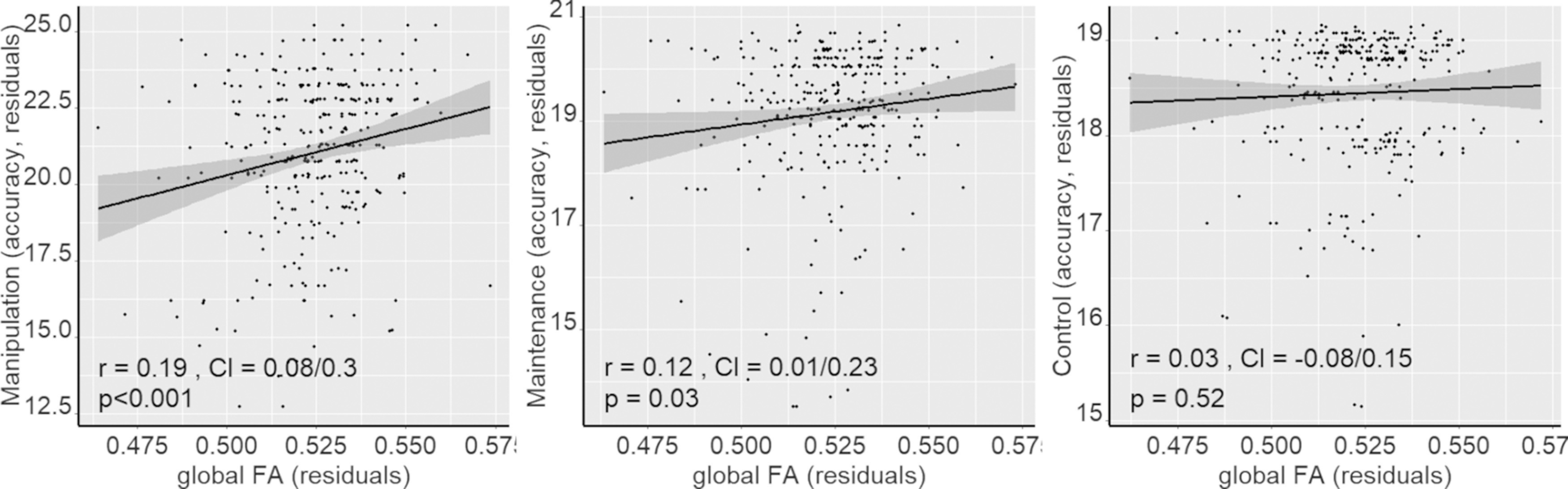
Positive relationship between mean FA across the entire skeleton and working-memory performance in manipulation (2 outliers removed, total *n* = 314), maintenance (7 outliers removed, total *n* = 309), and control conditions (10 outliers removed, total *n* = 306). Outliers are defined as FA or WM values >3.5 SDs from the mean. All the values are age- and sex-adjusted residuals. Confidence intervals (shaded area) are given by 2 × standard error.

FA was associated with WM performance for all three WM conditions in multiple white-matter tracts (Fig. 1). The voxels that showed significant positive associations with FA cover 36.5% (manipulation), 29.2% (maintenance), and 5.5% (control) of skeleton voxels, respectively. As can be seen in Figure 1, tracts with significant associations include the corpus callosum (genu, body, and splenium), internal capsule (anterior, posterior, and the retrolenticular part), external capsule, corona radiata (anterior, superior, and posterior), and superior longitudinal fasciculus. Figure 2 shows the correlation coefficients between mean FA across the entire skeleton and WM performance. All associations are independent of age and sex. Correlation coefficients among mean values of MD/RD/AD and working-memory performance are reported in Table 3.

**Table 3:** Correlation coefficients between MD/RD/AD and working-memory performance

	Manipulation		Maintenance		Control	
	*r*	*p*	*r*	*p*	*r*	*p*
MD	−0.15	0.01	−0.07	>0.05	−0.11	>0.05
RD	−0.16	0.005	−0.1	>0.05	−0.1	>0.05
AD	−0.09	>0.05	0.002	>0.05	−0.09	>0.05

Although the white matter–WM associations were stronger and more widespread in the high-WM load compared with the low-WM load condition (Figs. 1, 2), no significant differences among WM conditions were found. Since the load effects on WM performance were observed only in participants >55 years old, we repeated the analyses on this subsample, but found no load effect in these participants either.

**Figure 3.**
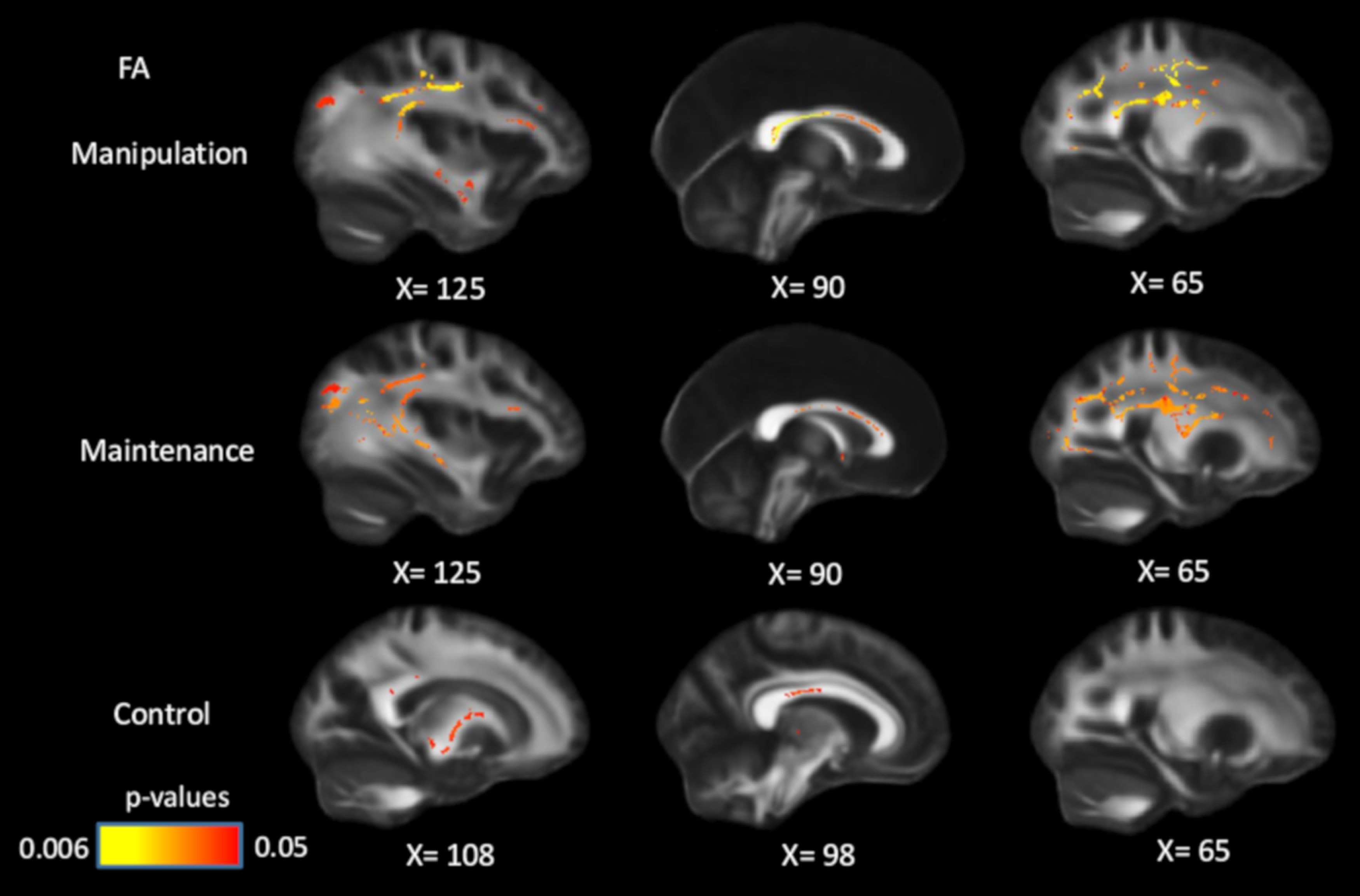
Positive associations between FA and WM performance across conditions, controlling for processing speed, age, and sex. After controlling for speed, the associations between WM and FA became less widespread, but remained significant in all three conditions. Significant voxels (*p* < 0.05) are overlaid on the T1-weighted image, with yellow color indicating lower *p* values. Coordinates are given according to the MNI152 template.

After controlling for processing speed, age, and sex in GLMs, the associations between FA and WM manipulation/maintenance became less widespread but remained significant in multiple white-matter tracts (Fig. 3), including corpus callosum, coronal radiata, superior longitudinal fasciculus, and posterior thalamic radiation. The FA–control associations largely disappeared after controlling for processing speed, but remained significant in parts of the corpus callosum (body and splenium) and internal capsule. These results suggest that the association between white matter and WM, especially in high WM-demanding conditions, is partly independent of individual differences in speed.

**Figure 4.**
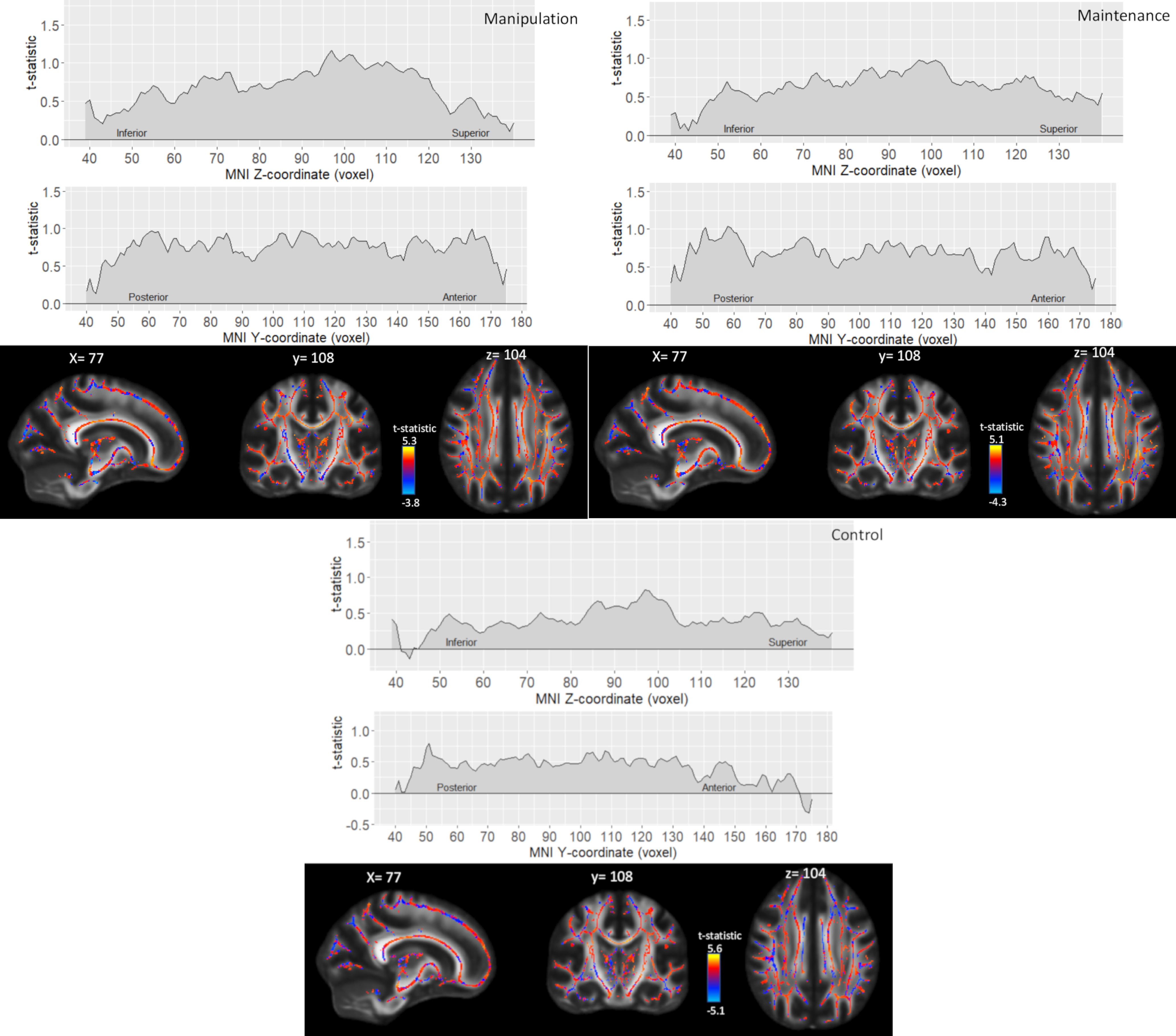
Spatial pattern of the associations between FA and manipulation, maintenance, and control conditions. Slice-by-slice profiles of mean *t* statistics in axial plane (top) and coronal plane (middle), and *t* statistics for the whole skeleton (bottom).

Slice-by-slice profiles of mean *t* values did not reveal stronger FA–WM associations in anterior compared with posterior brain regions in any WM condition, and the associations did not follow a superior–inferior gradient either (Fig. 4).

**Figure 5.**
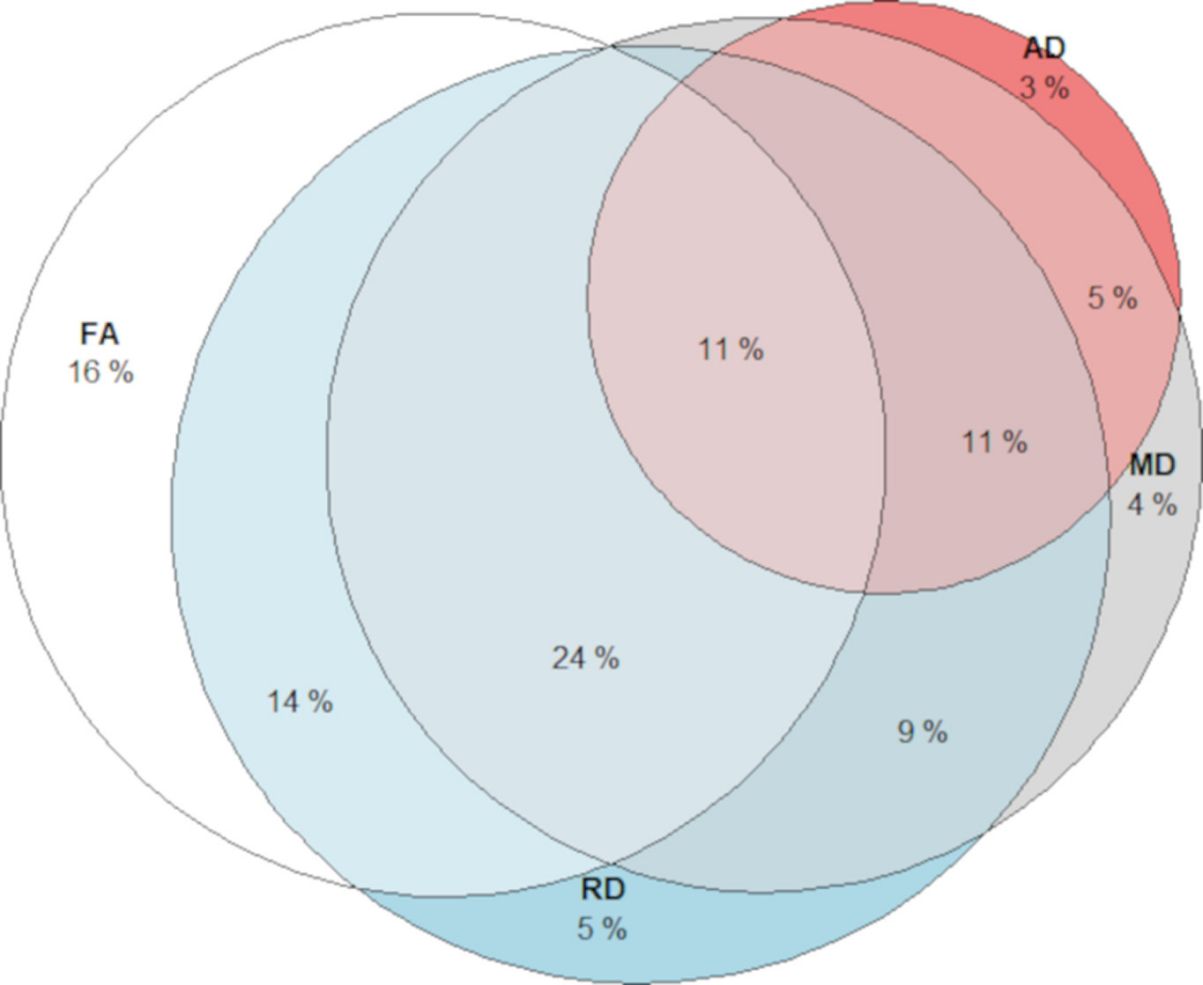
Overlap of voxels showing significant (*p* < 0.05) white matter–WM (manipulation) associations for each DTI measurement. Different segments illustrate the proportion of voxels showing the association between WM manipulation with FA only (white), MD (gray), RD (blue), and AD (red).

We present the patterns of white matter–WM manipulation associations for the DTI parameters in Figure 5. FA, MD, and RD showed more widespread associations with WM in the skeleton compared with AD. The most prevalent relationships were positive associations for FA, and negative association for MD and RD, which occupied 35% of all significant voxels in the skeleton.

### The association between age and WM performance is mediated by white-matter microstructure

**Figure 6.**
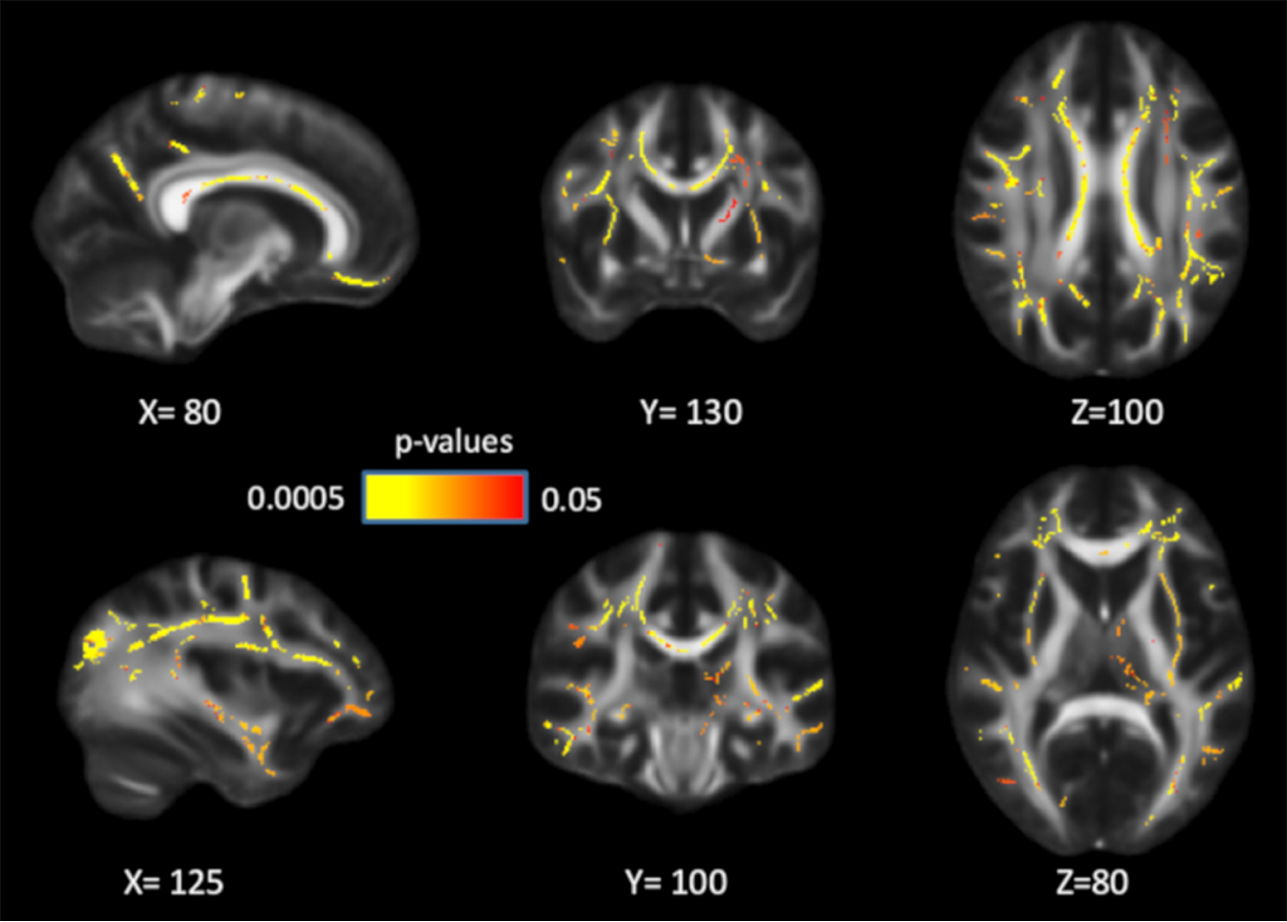
Mediation effects of FA on the association between age and working-memory performance (manipulation). Significant voxels (*p* < 0.05) are overlaid on the T1-weighted image, with yellow color indicating lower *p* values. Coordinates are given according to the MNI152 template.

Because there was no WM-load effect on FA–WM associations, we next focused on the manipulation condition to reduce the number of comparisons. We found that FA mediated the negative relationship between age and performance in the manipulation condition. This mediation effect was observed in 34% of the corpus callosum, 24% of the corona radiata, 38% of the posterior thalamic radiation, 10% of the external capsule, and 47% of the superior longitudinal fasciculus (Fig. 6). We then evaluated the mediation effects of FA within the significant voxels and found that the relationship between age and WM was largely attenuated (*t* = −8.99; CI = −0.12, −0.78; *p* < 0.001) after adjusting for individual differences in white-matter integrity (*t* = −7.38; CI = −0.11, −0.64; *p* < 0.001; 10.9% *r*^2^ drop).

### Genetic effects

**Figure 7.**
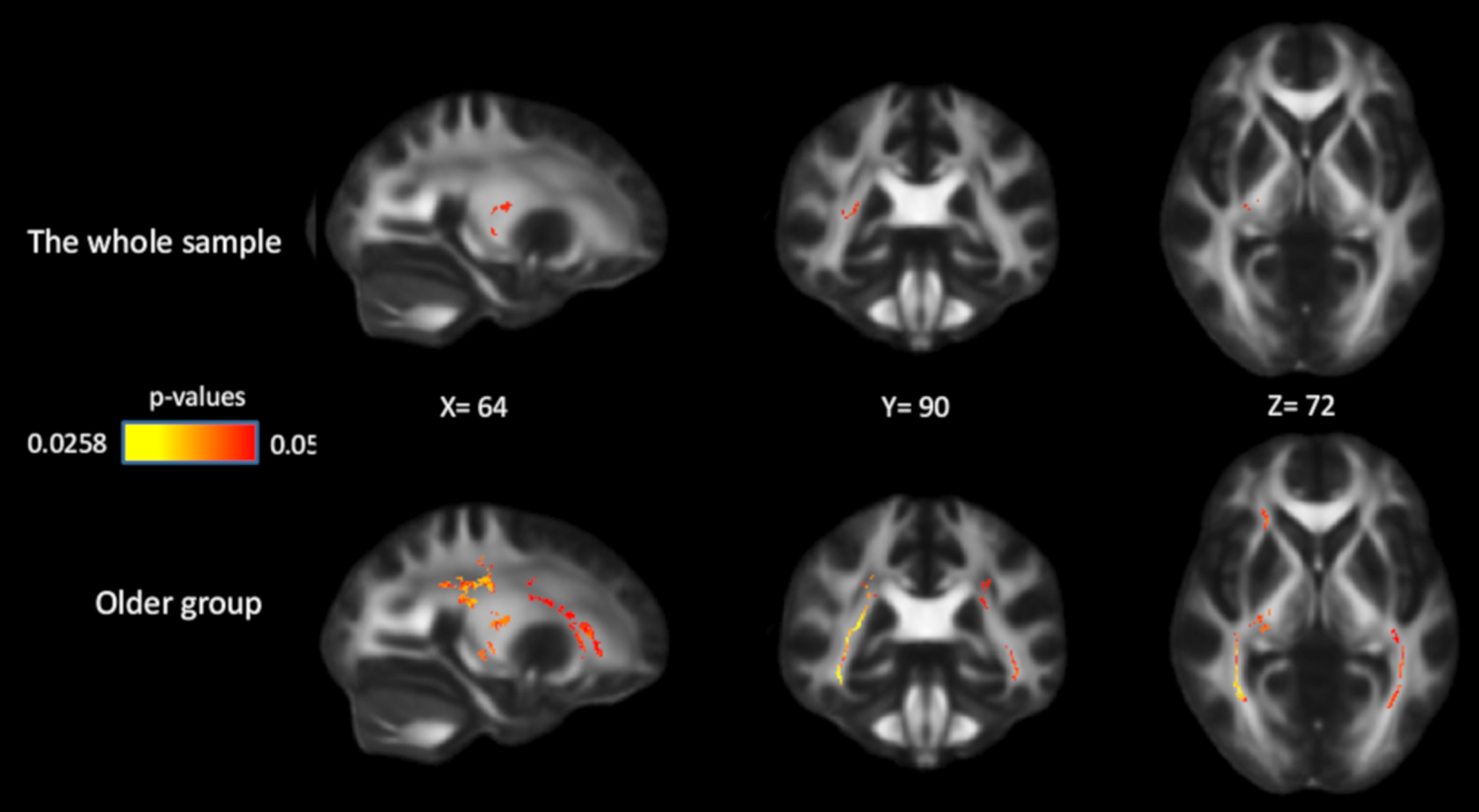
Effects of *COMT* on FA in the whole sample and in the older group. Significant voxels (*p* < 0.05) are overlaid on the T1-weighted image, with yellow color indicating lower *p* values. Coordinates are given according to the MNI152 template.

Compared with *COMT* ValVal carriers, ValMet heterozygotes had higher FA in several white-matter tracts, as follows: internal capsule, 12.7% of the whole skeletonized tract; corona radiata, 4.5%; posterior thalamic radiation, 14%; sagittal stratum, 12%; and superior longitudinal fasciculus, 4.9%. MetMet carriers had higher FA values than ValVal carriers, and lower FA values than ValMet heterozygotes, but these differences were not significant (lowest *p* values are 0.13 and 0.25 respectively). No significant associations were found between FA and the other two genetic polymorphisms. We further investigated the age × *COMT* interaction for FA and found that genetic effects were observed in the group of older adults only. Compared with ValVal carriers, ValMet carriers had higher FA in 25.7% of the internal capsule, 24.2% of the corona radiata, 42.1% of the posterior thalamic radiation, 66.4% of the sagittal stratum, and 26.5% of the superior longitudinal fasciculus in older adults (Fig. 7). In older adults, MetMet carriers had higher FA than ValVal carriers, and lower FA values than ValMet heterozygotes, but these differences were not significant (lowest *p* values are 0.06 and 0.1 respectively).

## Discussion

The current study investigated associations between white-matter integrity and WM using TBSS. White-matter integrity (FA/MD/RD) was associated with WM performance across all three WM loads. Associations were observed in multiple white-matter tracts, including corpus callosum, internal capsule, external capsule, corona radiata, posterior thalamic radiation, and superior longitudinal fasciculus. There was no evidence that the associations followed an anterior–posterior or superior–inferior gradient. These results indicate that WM might rely on connections of multiple regions across the whole brain. The associations were independent of age and sex, and remained partly significant after controlling for processing speed. Furthermore, white-matter integrity mediated the associations between age and WM, suggesting that compromised white-matter integrity partly accounts for less efficient WM processes in aging. Finally, limited, yet significant, effects of the *COMT* Val158Met polymorphism on FA were observed, which support the role of dopaminergic systems in white-matter integrity.

The finding that WM performance was positively associated with FA and was negatively associated with MD is consistent with past research linking white-matter integrity to various cognitive domains (O’Sullivan et al., 2001; Charlton et al., 2006; Grieve et al., 2007; Davis et al., 2009; Perry et al., 2009; Vernooij et al., 2009; Kennedy and Raz, 2009b; Gold et al., 2010). The current study adds new insights to this research field. Compared with previous work, we were able to demonstrate these white matter–WM associations in a large population-based sample using a voxelwise TBSS approach. As noted, a voxelwise approach should be more sensitive in detecting associations compared with an ROI approach. Indeed, associations between white-matter integrity and WM after controlling for age were observed across a major portion of the skeleton, compared with the smaller number of tracts found in previous ROI analyses (Kennedy and Raz, 2009a). The fact that the associations cover multiple white-matter tracts rather than being localized to specific pathways is also consistent with the notion that WM involves multiple interacting cognitive processes and requires dynamic cross talk among a large number of brain regions (Rottschy et al., 2012; D’Esposito and Postle, 2015; Eriksson et al., 2015; Liang et al., 2016; Salami et al., 2018).

Associations between white matter and WM were observed for the genu, body, and splenium of the corpus callosum. These patterns corroborate previous work (Grieve et al., 2007; Kennedy and Raz, 2009a) and point to a critical role for these regions in across-brain interhemispheric communication associated with WM processes (Avelar-Pereira et al., 2020). The associations demonstrated in the superior longitudinal fasciculus reflect that long tracts connecting frontal to more posterior regions within one hemisphere are important to WM. This is in line with fMRI work showing that frontoparietal regions are critical to higher cognitive functions, including WM (Kastner and Ungerleider, 2000; Rypma and D’Esposito, 2000; Curtis and D’Esposito, 2003; Chee and Choo, 2004; Collette et al., 2005; Koenigs et al., 2009). Moreover, in the current study, FA values in the superior longitudinal fasciculus were more strongly associated with WM in the manipulation condition than in the maintenance condition. Compared with manipulation, the maintenance condition taxes mainly short-term memory storage rather than switching and shifting. The observed patterns are thus consistent with previous findings showing a link between switching/shifting and white-matter integrity in the superior longitudinal fasciculus (Grieve et al., 2007; Perry et al., 2009), frontoparietal regions (Gold et al., 2010), and external capsule (Salami et al., 2018). Associations were also seen in the posterior thalamic radiation, which connects thalamus and visual cortex. These results are in agreement with findings showing a role of the ventral visual pathway in processing and storing information in WM (Ranganath et al., 2004).

Consistent with the known role of the striatum in WM functions (Cools et al., 2004; O’Reilly, 2006; Dahlin et al., 2008; McNab and Klingberg, 2008), we also found that white matter in the internal capsule was associated with WM performance. This particular region contains white-matter fibers connecting the lentiform and caudate nuclei, and fibers connecting the cortex and subcortical regions, such as caudate, putamen, and thalamus. Previous studies suggest that the PFC and the basal ganglia collaborate and serve a gatekeeping function in WM tasks. According to this view, the PFC maintains current goal-relevant information while the basal ganglia sends updating or shifting signals to the PFC to filter out irrelevant information and to update new WM representations (Frank et al., 2001; O’Reilly, 2006; O’Reilly and Frank, 2006; McNab and Klingberg, 2008). To our knowledge, this is the first study showing a link between white matter in internal capsule and WM, extending functional imaging studies by relating WM to structural connections within the basal ganglia, and between basal ganglia and cortical regions.

We found WM load effects on performance, especially for participants >55 years of age. However, although the white matter–WM associations were most pronounced in the most demanding condition, the associations were not significantly different when the three conditions were directly compared. This may be expected, given that the correlations in WM performance among the three conditions were high (*r* values = 0.64, 0.74, and 0.57, for manipulation vs maintenance, maintenance vs control, and manipulation vs control, respectively). Thus, the inclusion of all three conditions makes the unique variance that white matter can account for in each one relatively small. The lack of a load-dependent relation between white matter and performance likely reflects the fact that age-related degradation in white-matter integrity is not the only neurobiological mechanism of the lower performance in WM manipulation in older adults. For example, age-related reductions in gray-matter morphology (Chee et al., 2009; Evangelista et al., 2021) as well as age-related DA losses might also account for age-related decline in WM (Volkow et al., 1998), especially in high WM load conditions (Salami et al., 2019).

In addition to WM, previous findings also demonstrate significant associations between white matter and processing speed (Salami et al., 2012; Haász et al., 2013; Peters et al., 2014; Kuznetsova et al., 2016). Although processing speed and working memory are strongly related (Ackerman et al., 2002; Hertzog et al., 2003; Waters and Caplan, 2005), they may have partly separate functional and structural neural signatures (Ackerman et al., 2002; Chen and Li, 2007). We found that, after controlling for processing speed, associations with WM remained significant in multiple white-matter tracts, including corpus callosum, superior and posterior corona radiata, posterior thalamic radiation, and superior longitudinal fasciculus. These results suggest that the white-matter–WM link is partly independent of processing speed, and that these tracts might be critical for WM maintenance and manipulation. However, note that the number of significant voxels in anterior parts of the corpus callosum (genu), anterior corona radiata, external capsule, and superior longitudinal fasciculus decreased when controlling for processing speed. These findings indicate shared influences on processing speed and WM for these white-matter tracts. Current results also show that white-matter integrity has both unique and shared influences on WM and processing speed, especially in corpus callosum and superior longitudinal fasciculus.

Our analyses of overlapping voxels showed that the white matter–WM associations of the DTI measurements did not have the same spatial pattern, and that AD might be less sensitive compared with FA/MD/RD when it comes to WM performance. Past research shows that the impact of aging was stronger in RD than in AD (Burzynska et al., 2010; Madden et al., 2012). Similarly, our mediation analysis shows that age-related difference in WM might be more attributed to the age difference in RD compared with AD, especially in the body of the corpus callosum, corona radiata, and superior longitudinal fasciculus. RD has been linked to myelination, whereas AD might reflect axonal integrity (Heckel et al., 2015; Song et al., 2003; Sun et al., 2006, 2007). Thus, our results reflect that myelin degradation of these white-matter tracts might be more detrimental for WM compared with damage in axonal integrity in aging. However, the interpretation of the neurobiological mechanisms of DTI measurements is still inconclusive (Klawiter et al., 2011). One reason could be that most of the white-matter tracts contain crossing fibers. RD and AD measure the tensor (i.e., structural integrity) along and perpendicular to the major direction of the tract, which might be influenced by local crossing fibers. Additional research is needed to better understand the neural underpinning of DTI measurements.

Aging is associated with both WM decline and degradation of white-matter integrity (Hultsch et al., 1992; Park et al., 2002; Madden et al., 2012). The current finding that white-matter integrity mediated the association between age and WM corroborates past research targeting other aspects of cognition, such as processing speed (Salami et al., 2012; Peters et al., 2014), executive functions (Brickman et al., 2012), cognitive flexibility (Madden et al., 2009; Borghesani et al., 2013), and associative learning (Samanez-Larkin et al., 2012). Our results extend these findings to the domain of WM, using a large age-heterogeneous sample. Mediation effects were found in several of the major corticocortical tracts, including corpus callosum, external capsule, and superior longitudinal fasciculus, and projection fibers, such as corona radiata, and posterior thalamic radiation. Although these results are consistent with those of the stud by Charlton et al. (2008), note that, in that study, only mean MD values from a restricted number of consecutive slices were used for estimating white-matter integrity. Together, the current results add support to the notion that reduced white-matter integrity contributes to age-related impairments in cognition, possibly by interrupting coordinated processing and communication of distributed brain regions critical to WM.

Furthermore, we found a weak, but significant, effect on white-matter integrity of the *COMT* Val158Met allele, and this effect was mainly driven by older adults. The COMT enzyme is involved in extracellular degradation of synaptically released DA in the PFC (Matsumoto et al., 2003; Tunbridge et al., 2006, 2007). The Val158Met polymorphism in the gene encoding COMT modulates DA transmission in the PFC, with Val homozygotes having three to four times higher turnover rates than Met homozygotes (Lotta et al., 1995). Thus, prefrontal DA availability is lower in Val homozygotes, and higher in Met homozygotes. This polymorphism has been related to cognitive performance, especially executive function and working memory (Mattay et al., 2003; Nagel et al., 2008; Colzato et al., 2010), and influenced BOLD response in PFC during working memory (Mattay et al., 2003; Nyberg et al., 2014). Our findings suggest that the *COMT* gene might influence dopaminergic activity in the PFC, which subsequently leads to changes in brain axonal density and myelination. This is also in line with previous studies demonstrating a relationship between DA activity and myelination (Belachew et al., 1999; Hakak et al., 2001; Szeszko et al., 2005; Lindholm and Jazin, 2007). The fact that ValMet heterozygotes demonstrated higher FA compared with Val homozygotes in older adults could be partially driven by the fact that there was a larger number of ValMet heterozygotes compared with homozygotes (*n* = 35, 45, 86 for ValVal, MetMet, and ValMet, respectively), resulting in greater statistical power in the group of heterozygotes. We did not find a genetic effect on WM performance or on the white matter–WM association. This could be because of relatively low statistical power. Another reason could be that brain-based phenotypes, such as brain structure and function, might be more “proximal” and thus more sensitive to genetic variation than behavioral phenotypes (Green et al., 2008; Mattay et al., 2008; Rasetti and Weinberger, 2011; Meyer-Lindenberg, 2012). Consistent with the resource-modulation hypothesis (Lindenberger et al., 2008), the genetic effect was found primarily in older adults. However, the lack of age by gene interactions in the current study warrants caution in interpretation, as it might be attributed to the weak effect of any single polymorphism on white-matter integrity (Nyberg and Salami, 2014), which obviously decreases the power for detecting age by gene interactions.

Some limitations of the present work should be noted. Our study does not consider the influence of other macrostructural brain properties, such as gray-matter volume and white-matter hyperintensities. Decreased gray-matter volume and increased white-matter lesions are common in older adults (for review, see Salthouse, 2011). Given that more than one-third of the participants in our sample were >70 years of age, individual differences in other structural brain measures could have influenced the results. Also, the findings are based on cross-sectional data, and, therefore, we cannot make any causal inferences regarding true mediation effects of white-matter integrity on age-related WM deficits. TBSS also has its limitations (Jones and Cercignani, 2010; Bach et al., 2014). For example, as TBSS projects values for each voxel to the nearest skeleton location, voxels further from the center of the axons will be weighted less and may be artificially assigned to other tracts. This makes effects closer to the center of axons more easily detected and effects located in regions far from the axons less likely to be found (Schwarz et al., 2014). In addition, the tensor model used in TBSS cannot delineate tracts through crossing-fiber areas. Certain white-matter tracts may collapse on top of each other, and, therefore, potential effects in those brain regions cannot be detected using TBSS. Finally, because of the small effect sizes of single genes, the genetic effect of *COMT* observed in our study is rather weak and should be interpreted with caution. Further studies are needed to replicate these results and to investigate gene–gene interactions and relations among DA-related genes, white matter, and WM using larger samples.

In conclusion, we found that WM was associated with white-matter integrity in multiple tracts across the brain. The associations were partly independent of the effect of processing speed. In addition, age-related differences in white-matter integrity partly accounted for age-related WM deficits. Future longitudinal studies should address the issue of causality. Finally, consistent with our hypothesis, there was a weak genetic effect of the *COMT* Val158Met polymorphism on FA in old age, suggesting a link between DA activity and white-matter integrity.

## Synthesis

Reviewing Editor: Alexander Soutschek, Ludwig-Maximilians-Universitat Munchen Decisions are customarily a result of the Reviewing Editor and the peer reviewers coming together and discussing their recommendations until a consensus is reached. When revisions are invited, a fact-based synthesis statement explaining their decision and outlining what is needed to prepare a revision will be listed below. The following reviewer(s) agreed to reveal their identity: Eric Juarez.

The major contribution of this manuscript is its application of the voxel-based mediation technique for fractional anisotropy and aging. The reviewers’ impression of the dopamine-related gene finding is that it is tentative and exploratory, but provides evidence worth future study. Still, there are several concerns that need to be addressed in a revision.

1. Regarding the tractography analyses, there were a few places where the manuscript would benefit from clarification. For example, Line 177 (or earlier) please explain the acronym TBSS before introducing it (potentially include a citation as well e.g. Smith, S. M., Jenkinson, M., Johansen-Berg, H., Rueckert, D., Nichols, T. E., Mackay, C. E., et al. (2006). Tract-based spatial statistics: voxelwise analysis of multi-subject diffusion data. Neuroimage 31, 1487-1505).

Line 223: Why 5000 permutations? Others using this method use 10000 permutations. Does this make a difference?

Line 258-259: I don’t know whether it is customary for white matter papers, but it might be useful to pair the percentages of coverage with the structure.

2. Several aspects of the data analysis seem rather post-hoc and require a better justification. For example, for the genetic analyses the sample was split into two groups, but the authors do not describe how the threshold of 60 years was chosen. As the behavioral analysis suggests significant effects of working memory conditions for the 55+ age groups, maybe 55 years is a better threshold for splitting the sample into two groups? Are the genetic effects robust to using 55 years as threshold?

3. In the genetic analyses, AA (14 participants) and AG (93 participants) are combined and compared with GG (208 participants) carriers “so that the 2 groups have comparable sample sizes”. However, the sample sizes are still far from being comparable (107 versus 208 participants), so this does not seem like a good reason to combine the AA and AG groups. Again, it would be interesting to see whether the result patter is robust to comparing only AG and GG carriers (excluding the 14 AA participants).

Similarly, it is stated that there is a trend for MetMet carriers having higher FA than ValVal carriers, but the p-value of p=0.13 is too far from the statistical threshold to be considered as a trend. This also undermines the authors’ justification for combining MetMet with ValMet carriers, as they had argued that both MetMet and ValMet carriers showed significant differences relative to ValVal carriers.

4. Line 71-79: Please describe the associations in more detail--might be easier to follow this paragraph if they are described in more detail, like the resource modulation hypothesis at the end of the paragraph

5. Line 113: Based on the numbers provided I get 327 subjects. Check what the actual number of subjects is.

6. Line 279: Not sure you can claim especially in high WM demanding conditions since you didn’t find a load effect, or are you referring to the particular alphabet task compared to other WM tasks in the literature?

7. Table 1 includes reports demographics for the age categories 35-40 years, 45-50 years, etc. Is this a typo or did the study indeed include no participants falling into the categories e.g. 41-44 years or 51-54 years?

## Author Response

Dear Dr. Soutschek,

We are hereby re-submitting our manuscript entitled “White-matter integrity and working memory: Links to aging and dopamine-related genes.” with the previous title “White-matter integrity and working memory: Relationships to aging and dopamine-related genes”. We were pleased to see that you and the reviewers found merit in our work, and we thank the reviewers for their constructive comments. We believe that we have addressed all the issues raised, and that the quality of the manuscript has been improved by this revision. In this document, we detail all changes that were made. To enhance the readability of the response letter, the reviewers’ comments are in plain text, and our responses are in bold font.

We hope that you find the changes made to the manuscript to be satisfactory. Please let us know if you have any further questions.

Kind regards,

The authors

The major contribution of this manuscript is its application of the voxel-based mediation technique for fractional anisotropy and aging. The reviewers’ impression of the dopamine-related gene finding is that it is tentative and exploratory, but provides evidence worth future study. Still, there are several concerns that need to be addressed in a revision.

1a. Regarding the tractography analyses, there were a few places where the manuscript would benefit from clarification. For example, Line 177 (or earlier) please explain the acronym TBSS before introducing it (potentially include a citation as well e.g. Smith, S. M., Jenkinson, M., Johansen-Berg, H., Rueckert, D., Nichols, T. E., Mackay, C. E., et al. (2006). Tract-based spatial statistics: voxelwise analysis of multi-subject diffusion data. Neuroimage 31, 1487-1505).

Response:

We have now changed “TBSS” into “Tract-based spatial statistics (TBSS)” on P. 9, and added the following sentence: “We used TBSS (Smith et al., 2006) to analyze the FA/MD/AD/RD images.” as the first sentence in this section.

1b Line 223: Why 5000 permutations? Others using this method use 10000 permutations. Does this make a difference?

Response:

We agree with the Reviewer that a higher number of permutations will result in slightly more accurate p-values. However, 5000 permutations are more commonly used in this research area, and this is also the default setting in the FSL Randomize toolbox.

As can be seen in the figure (generated by Dr. Steven M. Smith, at the Oxford University Centre for Functional MRI of the Brain (FMRIB; https://www.jiscmail.ac.uk/cgi-bin/wa-jisc.exe?A2=FSL;7fef8d32.0803), if the true p-values are smaller than 0.04, 100% of the permutated p-values will be smaller than 0.05 for both 5000 permutations and 1000 permutations, and around 98% of the reported p-values will be smaller than 0.05 for 2500 permutations, which is still acceptable. If the true p-values are larger than 0.06, 100% of the reported p-values will be larger than 0.05 for both 5000 permutations and 1000 permutations, but around 98% for 2500 permutations. From this we conclude that conducting 5000 permutations is generally suitable for TBSS analyses.

To directly compare the differences of p-values in the current study, we reran the analysis of the associations between FA and WM for 5000 permutations and 10000 permutations. All results were replicated when 10000 permutations were used. For the voxels reported significant using 5000 permutations, 100% of those have p-values < 0.05 if permutated 10000 times. The differences of the p-values between 5000 and 10000 permutations range from -0.0003 to 0.003. This is the same for insignificant results, i.e., 100% of the voxels reported insignificant in 5000 permutations also showed P > 0.05 if permutated 10000 times. The differences of the p-values range from -0.0005 to 0.007. Thus, we believe that 5000 permutations are sufficient for controlling false positives in our study.

1c Line 258-259: I don’t know whether it is customary for white matter papers, but it might be useful to pair the percentages of coverage with the structure.

Response:

We present voxels that were significantly associated with WM performance for 3 WM conditions in Figure 1, and also describe the percentages of coverage for the white-matter tracts that were included. Also, to make it easier to follow, we revised the sentence on P.14:

"As can be seen in Figure 1, tracts with significant associations include the corpus callosum (genu, body, and splenium), internal capsule (anterior, posterior, and the retrolenticular part), external capsule, corona radiata (anterior, superior, and posterior), and superior longitudinal fasciculus.”

2. Several aspects of the data analysis seem rather post-hoc and require a better justification. For example, for the genetic analyses the sample was split into two groups, but the authors do not describe how the threshold of 60 years was chosen. As the behavioral analysis suggests significant effects of working memory conditions for the 55+ age groups, maybe 55 years is a better threshold for splitting the sample into two groups? Are the genetic effects robust to using 55 years as threshold?

Response:

We chose the cut-off of 60 years to separate younger and older individuals, as people at 60 years or above are commonly classified as older adults. However, we agree with the Reviewer that the threshold can be considered as somewhat arbitrary, as there are other studies defining older adults as 65 years and over, or 55 years and over. To address this issue, we re-analyzed the data using different cut-offs from 50 to 75 years. Because the genetic effects were only observed in older adults, the analyses were conducted in older adults only. For this analysis, the MetMet and ValMet groups were not combined into an anyMet group, which is further discussed in Question 3b.

The results patterns were identical no matter which cut-off was chosen, except for 70 and 75 due to small sample sizes. The white-matter tracts that demonstrated significant genetic influences using a cut-off of 60 years were also significant when other cut-offs were used. In the revised manuscript, we split the whole sample into {less than or equal to} 60 and > 60 age groups, because we wanted to ensure that the 2 age groups were approximately equal in sample size (n=34, 42, 73; and 35, 45, 86 for ValVal, MetMet,ValMet in younger and older groups correspondingly). We revised the Results section (P.16-17) accordingly:

"We further investigated the age × COMT interaction for FA, and found that genetic effects were observed in the group of older adults only. Compared to ValVal carriers, ValMet carriers had higher FA in 25.7% of the internal capsule, 24.2% of the corona radiata, 42.1% of the posterior thalamic radiation, 66.4% of the sagittal stratum, and 26.5% of the superior longitudinal fasciculus in older adults (Figure 7). In older adults, MetMet carriers had higher FA than ValVal carriers, and lower FA values than ValMet heterozygotes, but these differences were not significant (lowest p = 0.06 and 0.1 respectively).”

3a In the genetic analyses, AA (14 participants) and AG (93 participants) are combined and compared with GG (208 participants) carriers “so that the 2 groups have comparable sample sizes”. However, the sample sizes are still far from being comparable (107 versus 208 participants), so this does not seem like a good reason to combine the AA and AG groups. Again, it would be interesting to see whether the result patter is robust to comparing only AG and GG carriers (excluding the 14 AA participants).

Response:

This is indeed a good suggestion. We reanalyzed the data by excluding the A participants (n = 14). There was still no difference in white-matter integrity between the AG and the GG groups (lowest p-values: 0.59 for AG>GG; 0.46 for AG<gg). from="” this="” analysis="” it="” is="” evident="” that="” the="” pattern="” of="” results="” for="” ankk1="” polymorphism="” robust.="” however,="” we="” agree="” with="” reviewer="” motivation="” combining="” aa="” and="” ag="” groups="” may="” not="” be="” sufficient.="” therefore="” did="” combine="” groups,="” report="” demographic="” information="” 3="” genetic="” (aa="” gg)="” separately.<br="">3b Similarly, it is stated that there is a trend for MetMet carriers having higher FA than ValVal carriers, but the p-value of p=0.13 is too far from the statistical threshold to be considered as a trend. This also undermines the authors’ justification for combining MetMet with ValMet carriers, as they had argued that both MetMet and ValMet carriers showed significant differences relative to ValVal carriers.

Response:

We thank the Reviewer for raising this point and agree that we did not have enough motivation to combine MetMet with ValMet carriers. Thus, we decided to report the results for the three genetic group separately (P. 16-17), and discussed this result on P. 22. We now state that:

"The fact that ValMet heterozygotes demonstrated higher FA compared to Val homozygotes in older adults could be partially driven by the that fact that larger number of ValMet heterozygotes compared to homozygotes (n=35, 45, 86 for ValVal, MetMet, and ValMet respectively), resulting in greater statistical power in the group of heterozygotes.” (P. 22)

4. Line 71-79: Please describe the associations in more detail--might be easier to follow this paragraph if they are described in more detail, like the resource modulation hypothesis at the end of the paragraph

Response:

We are now reporting additional details about the genetic effects on white-matter integrity and WM functioning (P. 4). Hopefully, these revisions will make it easier to follow this part of the introduction. We now state that:

"For example, carriers of the COMT Met allele, which has been associated with higher DA levels in the prefrontal cortex (PFC; Lotta et al., 1995), had greater white-matter integrity (Papenberg et al., 2015), and higher efficiency in the dorsolateral PFC during WM performance (Nyberg et al., 2014) compared to Val carriers.”

5. Line 113: Based on the numbers provided I get 327 subjects. Check what the actual number of subjects is.

Response:

We thank the Reviewer for pointing this out. Some participants with both neuropsychiatric disorder and deviant brain morphology were not correctly reported in the previous version of the manuscript. We have now corrected these numbers (P. 5).

6. Line 279: Not sure you can claim especially in high WM demanding conditions since you didn’t find a load effect, or are you referring to the particular alphabet task compared to other WM tasks in the literature?

Response:

Indeed, we did not find evidence that the associations between white-matter and WM changed as a function of WM load. However, we found that WM performance in the manipulation condition was worse than that in the maintenance and control conditions, especially in 55+ age groups (Table 1). Thus, “high WM-demanding conditions” refers to WM manipulation and maintenance, as these 2 conditions were more demanding and linked to significantly worse performance compared to the control condition.

7. Table 1 includes reports demographics for the age categories 35-40 years, 45-50 years, etc. Is this a typo or did the study indeed include no participants falling into the categories e.g. 41-44 years or 51-54 years?

Response:

Thank you for raising this point. This is not a typo. The participant recruitment in this study (the Betula project) is based on an age-homogeneous, narrow age cohort (NAC) design, where chronological age was held constant within each cohort (i.e., participants were recruited at ages 35, 40, 45 etc.). Use of the NAC design decreases confounding effects introduced by different age cohorts, such as education and nutritional level (Rönnlund et al., 2005; Sternäng et al., 2008). This information is now provided in the manuscript (P. 5).</gg).>
